# A Study of Prognosis, Outcome, and Changing Tendency of Hospitalized AMI Patients in Beijing Third-Grade A-Level Traditional Chinese Medicine Hospitals from 1999 to 2008

**DOI:** 10.1155/2012/837194

**Published:** 2012-03-22

**Authors:** Ju-Ju Shang, Hui Shi, Qi Zhou, Wei Gao, Hong-xu Liu

**Affiliations:** ^1^Beijing Traditional Chinese Medicine Hospital, Capital Medical University, Dongcheng, Beijing 100010, China; ^2^Cardiovascular Department, Beijing Traditional Chinese Medicine Hospital, Capital Medical University, Beijing 100010, China

## Abstract

*Objectives*. To survey and analyse the prognosis, outcome, and changing tendency of the Acute Myocardial Infarction (AMI) patients in Beijing third-grade A-level Traditional Chinese Medicine (TCM) hospitals. *Methods*. We collected the clinical datum of hospitalized AMI patients in Beijing 6 TCM hospitals from January 1999 to December 2008 and then analysed the clinical datum. *Results*. (1) The mean age of patients had showed a slowly rising tendency during this ten years. The patients who had previous history of cerebrovascular diseasea and multiple comorbidities had increased year by year. (2) The rate of reperfusion therapy, revascularization and standardized using of drug, and usage of TCM of AMI patients presented a significant increasing tendency in these hospitals. (3) The proportion of AMI patients combined with cardiac arrhythmia and heart failure had decreased significantly. (4) The AMI mortality presented a decreasing tendency in 10 years. *Conclusions*. The AMI patients in Beijing TCM hospitals had their own unique clinical features, and it can improve their prognosis by combined therapy of Western Medicine and Traditional Chinese Medicine.

## 1. Objectives

Cross-sectional registry was used to collect the hospitalized AMI patients' clinical datum in third-grade A-level TCM hospitals from 1999 to 2008 in Beijing, so as to dynamically analyse their clinical characteristics in recent 10 years and provide preliminary epidemiological datum of standardized treatment in TCM hospitals.

## 2. Participants

### 2.1. Source of Cases

The subjects we enrolled were hospitalized patients suffering from AMI in Beijing Third-grade A-Level TCM hospitals from January 1, 1999 to December 31, 2008. We totaly recovered 2056 case report form (CRF) handbooks, and by screening there were 2053 within registered. See Table 1.

### 2.2. Inclusion Criteria

(1) All participants in the study hospital's records and statistics room in the computer management system of the international classification of diseases (ICD) were coded for AMI; (2). All these patients were diagnosed as AMI by reference of the Chinese society of Cardiology, Chinese Journal of cardiovascular medicine, Editorial Committee of Chinese Circulation Journal jointly developed the* Guidelines for the Diagnosis and Treatment of Acute Myocardial Infarction *(2001) [1] (the following referred to as the* Guideline*). (3) The information of research-related syndromes of Chinese medicine were filled or by adding completely then could be used. Conforming to the above three criteria can be inclused into the survey.

### 2.3. Exclusion Criteria

(1) Old AMI patients. (2) Patients'. TCM syndrome information was seriously uncomplete and unable to complement. In accordance with neither of the two criteria, it would be been exclused. 

## 3. Methods

First of all the Capital Research Foundation for Medical Development group designed the CRF handbook which included the general characteristics, hospitalized therapeutic conditions and the prognosis, and outcome of AMI patients. Then we gave the CRF to the doctors of cardiovascular department of each hospital. In every hospital, elected cases were filled according to the actual situation by the first doctor who had been trained.  About every six months, all the cases of cooperate units were summarized to the cardiovascular department of Beijing TCM Hospital, then the task group inspected the datum and eliminated invalid datum.

Next the datum was entered to the survey database which was established based on ACESS 2000 by China Department of Epidemiology, Capital Medical University Beijing Anzhen Hospital. The datum was entered by the trained persons.  We used double datum entry, consolidated datum, and then checked error by the logic difference method. Statistical software SPSS15.0 (SPSS Base15.0S, PN: 32119001, SN: 5045602, L20080005) was used in descriptive analysis for general datum, measurement datum was analyzed by *F* test and *Q* test, and two sets of measurement datum were analyzed by *T* test; categorical data was analyzed by *X* ^2^ test.

## 4. Results

### 4.1. General Characteristics

#### 4.1.1. Age

**Figure 1:**
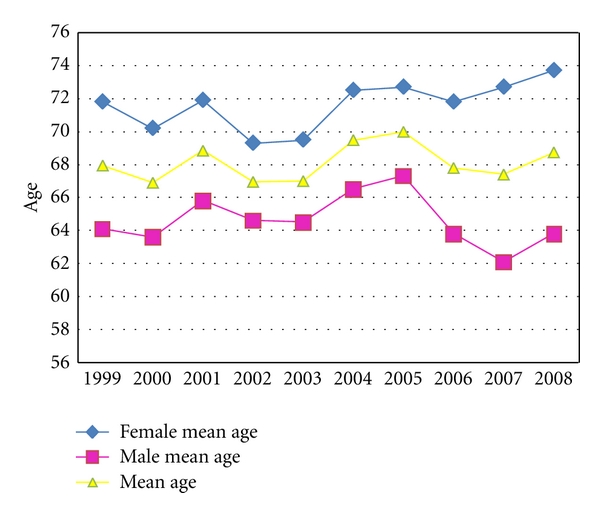
The age change trend of AMI patients in third-grade A-level TCM hospitals in Beijing from 1999 to 2008.

A total of 2053 AMI patients were entered, of these, 2015 cases had clearly recorded the age of onset, the minimum age was 18 years old, the maximum age was 105 years old, and the mean age was 67.37 ± 12.10 years old. The average onset age showed a slowly rising tendency from 1999 to 2008. 1316 cases were male patients, 737 cases were female patients, and the gender ratio was 1.8 : 1. For the changing tendency of AMI patients' see Figure 1.

#### 4.1.2. The History and Risk Factors

**Figure 2:**
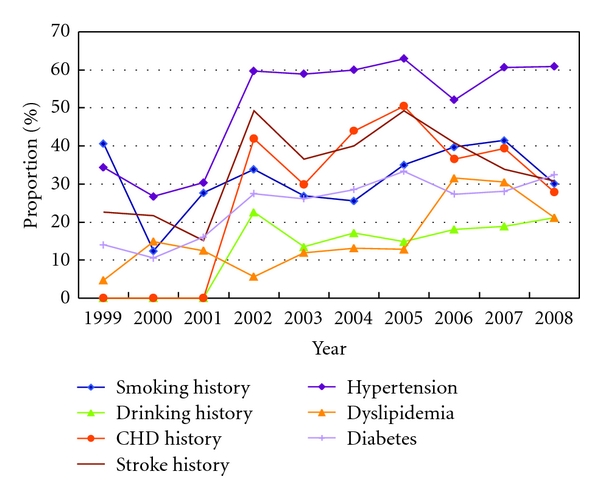
The risk factors change trend of AMI patients in third-grade A-level TCM hospitals in Beijing from 1999 to 2008.

There were total 680 patients with a clear history of smoking and 299 cases with a history of clear drinking. Patients with a history of smoking drinking in the proportion of overall show a slow ascending tendency. In 638 cases with clear history of coronary heart disease, the proportion had decreased. 695 patients had a clear stroke history, the proportion had gone up. 1130 patients combined with hypertension, and 438 patients had dyslipidemia, 542 patients combined with diabetes mellitus; the proportion of those patients to the overall was in a upward trend from 1999 to 2008 (see Figure 2).

#### 4.1.3. AMI Status

**Figure 3:**
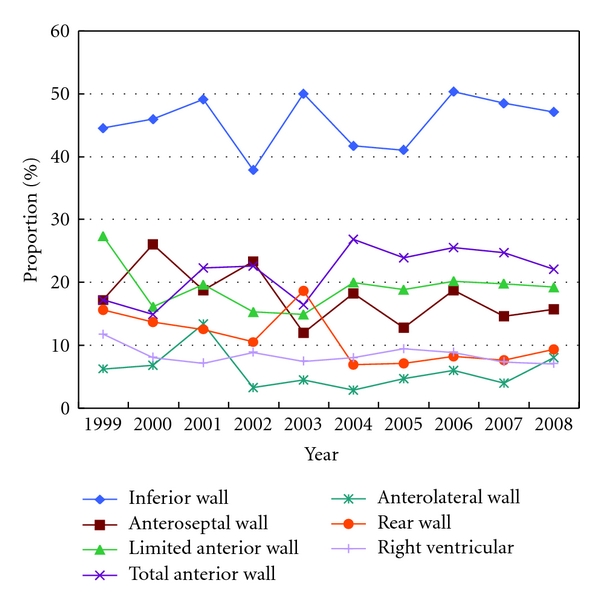
The proportion of MI location of hospitalized AMI patients from 1999 to 2008.

The total anterior wall myocardial infarction (MI) cases was 1324, and the proportion had no obvious change; the rear wall MI cases were 1147, right ventricular MI cases were 170, and the proportion of inferior wall and right ventricular MI decreased slowly. Clear ST segment elevation cases were 1287; clear Q wave MI cases were 1231; 1616 cases were the first MI, 243 cases were second MI, 16 cases were third MI, and 178 cases were unspecified (see Figure 3).

A total of 656 patients underwent coronary angiography, accounted for 31.95 percentage of the observed cases; patients undergoing coronary angiography increased year by year from 2001 to 2008.

### 4.2. Hospitalized Therapeutic Conditions

#### 4.2.1. Reperfusion Therapy

In 2003, the usage rate of reperfusion therapy was the lowest (20.97%), while in 2008, it had gone up to 53.80%, which presented an upward tendency from 1999 to 2008. The usage rate of intravenous thrombolysis therapy was the lowest in 2008 (1.28%), while in 2008, it was the highest up to 28.57%, which presented a downward tendency from 1999 to 2008.

There was no emergency PCI patient in 1999 and 2000. The rate of underwent emergency PCI was the lowest in 2008 (1.79%), while in 2006, it was the highest up to 24.82%, which presented an upward tendency from 1999 to 2008. The rate of underwent early reperfusion was the lowest in 1999 (23.44%), while in 2007, it was the highest up to 37.80% which presented an upward tendency from 1999 to 2008.

There was no patients undergoing CABG in 2000, 1999, 2007, and 2008, CABG patients have the highest proportion (1.61%) in 2002, and from 1999 to 2008 there had been no obvious regularity.  There was no patients undergoing remedial PCI in 1999 and 2001. The remedial PCI patients covered the lowest proportion in 2003 as 1.49% and the highest proportion in 2008 as 8.01%, which presented an ascendant tendency from 1999 to 2008.  There was no patient undergoing selective PCI in 1999 and 2000; the selective PCI patients covered the lowest proportion as 0.89% in 2001 and the highest as 20.51% in 2008, which also presented an ascendant tendency from 1999 to 2008. Specific reperfusion form is shown in Figure 2.

#### 4.2.2. Oral Drug Therapy

Western medicine intervention: the usage rate of drugs followed the *Guideline* recommendations from 1999 to 2008 which were the following: aspirin was 86.64%, clopidogrel was 37.91%, nitrate was 88.91%, beta blockers were 67.84%, angiotensin-converting enzyme inhibitors (ACEIs) and angiotensin receptor blocker (ARB) together were 77.75%, low molecular weight heparin was 82.38%, and adjustable lipid drug was 54.37% see Figure 3.

**Figure 4:**
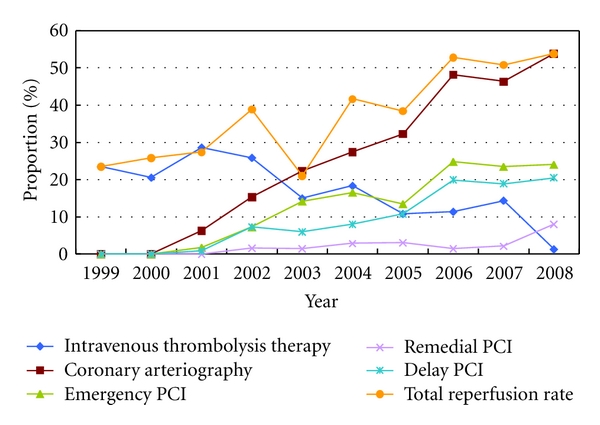
the reperfusion therapy conditions of AMI patients in third-grade A-level TCM hospitals in Beijing from 1999 to 2008.

The usage rate of other drugs: from 1999 to 2008 the usage rate of these drugs which were not recommended by the *Guideline* was the following: unfractionated heparin was 14.4%, antiarrhythmic drug was 16.1%, CCB was 23.4%, diuretic was 39.9%, digitalis cardiac drug was 16%, GIK was 20.4%. See Figures 4 and 6.

#### 4.2.3. The Intervention's Conditions of Chinese Medicine

**Figure 5:**
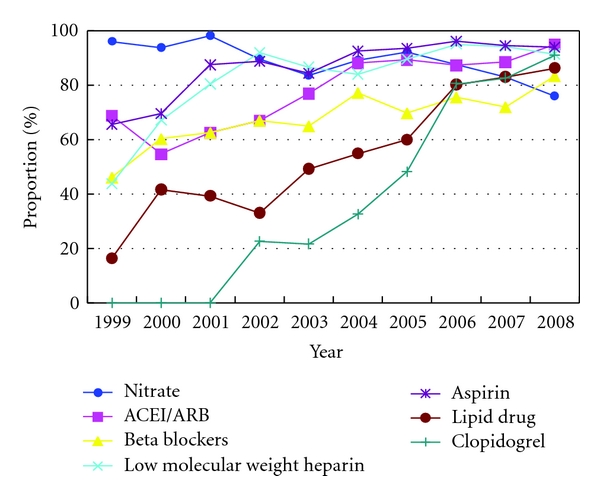
The status of oral drug usage followed the *Guideline *recommended to AMI patients in Third-grade A-Level TCM hospitals in Beijing from 1999 to 2008.

**Figure 6:**
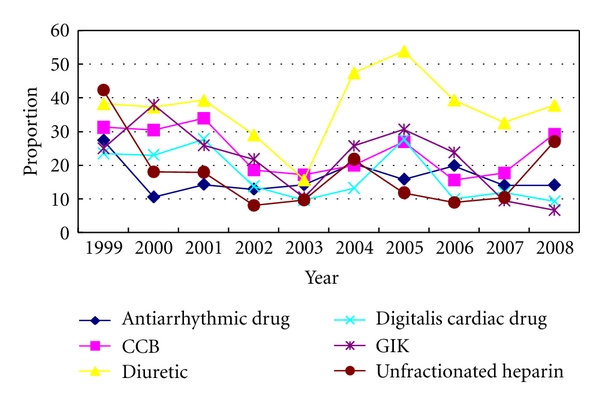
The rate of oral drugs which were not recommended by the* Guideline* to AMI patients in Third-grade A-Level TCM hospitals in Beijing from 1999 to 2008.

**Figure 7:**
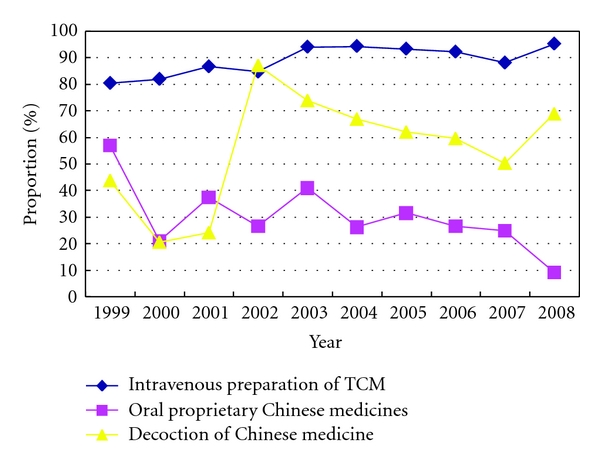
Chinese medicine intervention in AMI patients in Beijing Third-grade A-Level hospitals from 1999 to 2008.

From 1999 to 2008,  within 2053 patients, 1851 patients have used intravenous preparation of Chinese medicine in TCM hosptitals, and the total usage rate was 90.2%, which presented a tendency of escalation. 400 patients used oral proprietary Chinese medicines, and the general usage rate was 19.5%, which presented a decline in general from 1999 to 2008. 1056 patients using oral decoction, the total usage rate was 51.4%, and the usage rate of decoction of Chinese medicine was increasing in general from 1999 to 2008. The changing tendency of Chinese medicine intervention was shown in Figures 5 and 7.

### 4.3. Complications

Arrhythmia, heart failure, and cardiogenic shock are the common clinical complications of AMI patients, and are also the major cause of death. Based on the datum of 10 years in Beijing third-grade A-level TCM hospitals, the proportion of patients complicated by arrhythmia had dropped from 42.91% in 1999 to 24.04% in 2008, presented a fluctuating downward tendency; the proportion of patients complicated by heart failure had dropped from 60.16% in 1999 to 41.03% in 2008, and presented a significant downward tendency; patients complicated by cardiogenic shock had no obvious changes in the proportion.

### 4.4. Mortality

This survey showed that the mortality of AMI patients presented a fluctuating tendency of decline from 1999 to 2008 in Beijing Third-grade A-Level TCM hospitals, the main death due to cardiogenic death. With the increase of age, AMI's mortality gradually increased, and the hospitalized mortality of patients older than 75 years old was up to 22.91%.

Female AMI patients' hospitalized mortality was higher than that of male, which was 19.15%.

The survey also showed that the mortality of patients accompanied by arrhythmia, heart failure was significantly higher than in those without the complications (22.5% versus 8.8%, 37.8% versus 4.4%), and especially patients complicated with cardiogenic shock had a high mortality which was upto 56.9%.

## 5. Discussion

AMI is a serious cardiovascular disease which is hazard to human health and is the leading cause of death worldwide. In recent years, with the standardization of early reperfusion and drug treatment AMI mortality declined but it is still a high-mortality disease.

### 5.1. Clinical Characteristics

This survey presented that the average age of AMI patients was 67.37 years old, the patients older than 65 years old account for 64.40% of all observed cases, and the male to female ratio was 1.8 : 1.

A 26-year follow-up survey showed that the incidence of AMI of population aged 35 to 84 years old, for males was 71‰, for women, was 22‰. For the group from 55 to 64 years old, the incidence for male and female was  91‰  and  25‰, it were 119‰  and 51‰  in the 65*∼*74 years old group and; was 168‰  and  90‰  in the 75*∼*84 age group [2].

Compared with AMI patients on the study which was also sponsored by our study group to survey the therapeutic situations of TCM hospitals and Western medicine hospitals in Beijing in 2005 [3], we found that AMI patients in thrid-grade A-level TCM hospitals in Beijing area tended to be older, and the female proportion was much higher in TCM hospitals than that in western medicine hospitals.

### 5.2. Therapeutic Conditions

 Reperfusion therapy has become the most important means of treatment on AMI. Based on the datum of 10 years, the proportion of reperfusion therapy showed rising,  the proportion of intravenous thrombolytic treatment had descended, all these presented the whole reperfusion technology, and levels rose apparently in TCM hospitals in Beijing.

*The usage rate of drugs recommended by Guideline presented obviously increased, while the usage rate these drugs not recommended by Guideline presented a fluctuated decline, which prompted the gap between Beijing Third-grade A-level TCM hospitals and the Guideline demand narrowed gradually.*

In the development of China Traditional Chinese medicine had been playing an important role in preventing and treating all kinds of diseases. In the treatment of AMI, Chinese medicine also plays its role. This survey showed that Chinese medicine intravenous preparations currently have been widely used in clinically; this may be related to their convenient usage and the effective component being relatively single. Other several studies of our study group all show that Chinese medicine intravenous preparations can lower the mortality [3–6].

### 5.3. Complications

Arrhythmia, heart failure, and cardiogenic shock are the common clinical complications of AMI patients and are also the major cause of death. The datum showed the proportion of patients complicated by arrhythmia and heart failure presented a significant downward tendency during this 10 years in Beijing third-grade A-level TCM hospitals. All these showed that TCM hospitals in Beijing area have made unceasing progress in the treatment concept, treatment means, and so on, with the early reperfusion level rising ceaselessly and Chinese medicine vein preparation widely used clinically in AMI patients, patients can receive timely and effective treatment, and hospitalized complication will decrease gradually.

### 5.4. Mortality

The mortality of AMI correlates to age, gender, and complications. Our survey showed that AMI patients who had a history of stroke and coronary heart disease, combined with hypertension, diabetes mellitus patients and many hospitalized history had a relatively higher mortality, considering related to the factors as combined with more disease, relatively complex and worse vascular condition.

Arrhythmia, heart failure, and cardiogenic shock are common complications in patients with AMI; AMI complications and mortality are also highly correlated. The survey also showed that the mortality of patients accompanied by arrhythmia, heart failure was significantly higher than in those without the complications, and especially patients complicated with cardiogenic shock had a high mortality which was up to 56.9%.

## 6. Conclusions

Through this survey, we found patients who suffered from AMI in TCM hospitals in Beijing area were mainly old patients and the female patients were more than those in Western Medicine hospitals, and they had more complications. Our other studies have shown that [7–9] elder AMI patients either in clinical features, combined with risk factors, treatment, or on long- or short-term prognosis were significantly different from young patients, and especially for older female patients, it was different from male patients.

Traditional Chinese medicine had been playing an important role in preventing and treating all kinds of diseases for Chinese people. In the treatment of AMI, Chinese medicine also plays its role. Integrative medicine treatment, combining TCM and conventional medicine, has been the most representative characteristic for AMI. However, the potential benefit of integrative medicine therapy in improving AMI prognosis is still under studying. Our study showed that, by combined therapy of Western Medicine and Traditional Chinese Medicine, the mortality of AMI patients in Beijing TCM Hospitals presented a decreasing tendency, which presented that the integrative medicine might have potential benefit for AMI patients.

## Figures and Tables

**Table 1:** List of hospitals participating in the survey.

Hospital name	Cases	Proportion (%)
Beijing TCM Hospital	479	23.33
Xi Yuan TCM Hospital	584	28.45
Dong Fang TCM Hospital	456	22.21
Guang, anmen TCM Hospital	248	12.08
Dongzhimen TCM Hospital	204	9.94
Wangjing TCM Hospital	82	3.99
